# Nutrition and Cardiovascular Health

**DOI:** 10.3390/ijms21072284

**Published:** 2020-03-26

**Authors:** Paramjit S. Tappia, Heather Blewett

**Affiliations:** 1 Asper Clinical Research Institute & Office of Clinical Research, St. Boniface Hospital, Winnipeg, MB R2H2A6, Canada; 2 Agriculture and Agri-Food Canada, Winnipeg, MB R2H2A6, Canada; hblewett@sbrc.ca

There is unequivocal experimental, epidemiological and clinical evidence demonstrating a correlation between diet and increased risk of cardiovascular disease (CVD). While nutritionally poor diets can have a significant negative impact on cardiovascular health, dietary interventions with specific nutrients and/or functional foods are considered cost-effective and efficient components of prevention strategies. It has been estimated that nutritional factors may be responsible for approximately 40% of all CVD [1]. Indeed, in one of the seminal studies conducted on modifiable risk factors and heart health (the INTERHEART study), >90% of all myocardial infarctions were attributed to preventable environmental factors with nutrition identified as one of the important determinants of CVD [2]. There is increasing public interest and scientific investigation into establishing dietary approaches that can be undertaken for the prevention and treatment of CVD. This Special Issue provides an insight into the influential role of nutrition and dietary habits on cardiovascular health and disease as well as the therapeutic and preventive potential of novel nutraceuticals and specific nutrients. Fourteen outstanding papers, from experts in the field, provide a broad range of contributions detailing various aspects of nutrition and cardiovascular health as well as highlighting possible mechanisms of beneficial action.

Casas et al. [3] have reviewed the role of overall nutrition, specific nutrients, foods and dietary practices in relation to cardioprotection and prevention of CVD. This review Furthermore describes some of the mechanisms in the cardioprotective properties of individual nutrients, foods and nutritional patterns.

This Special Issue contains two reviews on the effects of long-chain polyunsaturated omega-3 fatty acids on CVD. Innes and Calder [4] provide a review of the literature on the use of marine derived omega-3 (n-3) fatty acids (eicosapentaenoic acid and docosahexaenoic acid) with a focus on primary and secondary prevention of CVD, along with a discussion of the potential mechanisms for their effects. Goel et al. [5] discuss the inconsistencies regarding the cardioprotective effects of fish and fish oils. Although many experimental studies and some clinical trials have documented the benefits of fish oil supplementation in decreasing the incidence and progression of atherosclerosis, myocardial infarction (MI), heart failure (HF), arrhythmias and stroke, recent large-scale clinical studies have failed to demonstrate any benefit on cardiovascular outcomes and mortality. This is an area of investigation that needs some refinement in order to fully understand the beneficial effects of fish oils and n-3 polyunsaturated fatty acids in general. Indeed, we have Furthermore reported inconsistencies in outcomes of CVD management in some trials with n-3 fatty acids [6,7].

Micronutrient deficiency is present in HF and is associated with adverse clinical signs and symptoms. Indeed, a pathophysiological role as well as prognostic value has been ascribed to micronutrient deficiency. In this regard, Cvetinovic et al. [8] review evidence that demonstrate a correction/normalization of micronutrient status is linked to a concomitant improvement in physical performance and quality of life in HF. The Special Issue Furthermore includes articles on specific micronutrients. Globally, vitamin D deficiency is highly prevalent and has been linked to many non-communicable diseases, but the role of vitamin D in the pathogenesis of HF remains to be defined. Roffe-Vazquez [9] discuss the molecular mechanisms involved in inflammatory processes, remodeling, fibrosis and atherosclerosis in humans due to a deficiency in vitamin D, through in vitro and animal experimentation. Furthermore, by employing human cardiac microvascular endothelial cells and a rat model of isoproterenol-induced fibrosis, Lai et al. [10] specifically report that vitamin D may, in fact, reduce the development of fibrosis. Although more research is required, vitamin D could be part of a prevention strategy for individuals at risk of HF.

The role of magnesium (Mg) deficiency as a risk factor of arterial hypertension is not completely known. Kostov and Halecheva [11] describe the many benefits of Mg and discuss the role of Mg deficiency in increasing the risk of atherosclerosis, endothelial dysfunction and arterial stiffness, which all contribute to hypertension. Thus, healthy dietary practices that include the recommended amounts of Mg may constitute a nutritional approach for normal blood pressure.

Nutraceuticals and functional foods have established efficacy for the treatment and/or prevention of adverse human health. Among these natural health products is American Ginseng. Parikh et al. [12] describe the cardioprotective effects of American Ginseng root extract (GBE) using a rat model of congestive heart failure due to MI induced by coronary artery ligation. Although treatment with GBE did not improve cardiac remodeling and function, attenuation of oxidative stress and TNFα was seen. The reduction of TNFα to below baseline levels was suggestive of the use of GBE as a prophylactic or as a preventive adjunct for cardiovascular disease. Jakovljevic et al. [13] have examined the potential health benefits of Aronia melanocarpa extract (SAE) in a rat model of metabolic syndrome (MetS). SAE was found to lower blood pressure and improve cardiac function. It is noteworthy that SAE improved glucose tolerance, liver damage and reduced oxidative stress. From the evidence provided, nutritional supplementation with SAE may potentially exert cardioprotective effects in patients with MetS. Cocaine is a potent stimulant drug that disrupts the electrical signals of the heart, increases blood pressure, heart rate, and the occurrence of heart-related fatal events including sudden cardiac death. In the review by Kim and Park [14] the cellular and molecular mechanisms of cocaine on the cardiovascular system are described. While there is unequivocal information on the adverse acute effects of cocaine on the heart (electrophysiological abnormalities, arrhythmia, and acute MI), the data on the chronic effects of cocaine on the vascular system (coronary artery disease (CAD) and/or subclinical atherosclerosis) are less clear and inconsistent. However, interestingly, chronic effects of cocaine are more likely in individuals with higher CAD risk and with deleterious health choices and behaviour.

Extracellular matrix (ECM) remodeling and fibrosis are key players in HF. Although some experimental studies have reported that nutraceuticals can diminish cardiac fibrosis, clinical data are conflicting. Jahan et al. [15] detail the molecular mechanisms involved in the control of fibroblast activation. Specifically, the role of Zeb2 transcription factor which could serve as an effective target for the attenuation/prevention of cardiac fibrosis is discussed and the potential of specific nutraceuticals described. Lim et al. [16] describe the current understanding of α-Klotho, a protein with anti-aging properties, as a potential therapeutic against age-associated vascular abnormalities. From the evidence presented in this review, it is conceivable that Klotho-based interventional trials could be initiated and yield important data for the effective clinical use of α-Klotho in several pathophysiological conditions including chronic kidney disease, cancer, diabetes and HIV infection where age-related vascular alterations are implicated. High sodium intake has been shown to have a positive relationship with death [17]. Paczula et al. [18] discuss the role of endogenous cardiotonic steroids in mediating salt-induced hypertension and organ damage. Compelling lines of evidence are provided in the link between high-salt diet and organ damage and thus it can be inferred that control of dietary salt is of critical importance in the prevention and/or risk reduction. Irisin has been characterized as a myokine that has been linked to insulin resistance, obesity and other non-communicable adult diseases. However, information regarding the role of irisin in childhood and early adulthood is inconsistent. In this regard, Elizondo-Montemayor [19] discusses the potential role of irisin in cardiovascular and metabolic health and disease in the pediatric population.

Taking all contributions into account it is clear that nutrition plays an important role in cardiovascular health and disease. In general, nutrients exhibit a diverse range of properties including anti-oxidant effects, anti-inflammatory actions, modification of signal transduction mechanisms, as well as metabolic, molecular and membrane actions. Although the cardioprotective effects may not be due to a single nutrient, but a balanced and varied diet of food items that can provide different benefits may prove to be key to cardiovascular health. We are grateful to all contributors, who are highly regarded and well-recognized in their respective field of interest. They have helped to generate a valuable issue of the journal that is very much special. It is hoped that experts within the field and those that have a general interest in nutrition and human health will find the information presented in this Special Issue, educational and insightful to prompt further investigation and advancement in understanding into the essential role of nutrition in cardiovascular health and disease treatment and/or prevention.
